# Superiority of systemic bleomycin to intradermal HOCl for the study of interstitial lung disease

**DOI:** 10.1038/s41598-023-47083-y

**Published:** 2023-11-23

**Authors:** Arina Morozan, Sydney Joy, Utako Fujii, Richard Fraser, Kevin Watters, James G. Martin, Inés Colmegna

**Affiliations:** 1 Meakins Christie Laboratories, McGill University Health Centre and McGill University, Montreal, QC H4A 3J1 Canada; 2 The Research Institute of the McGill University Health Centre, McGill University, 1001 Decarie Blvd, Office # EM2-3238, Montreal, QC H4A 3J1 Canada; 3 Division of Pathology, McGill University Health Centre, Montreal, QC Canada; 4 Division of Rheumatology, McGill University Health Centre, McGill University, Montreal, QC Canada

**Keywords:** Autoimmunity, Respiration

## Abstract

Systemic sclerosis (SSc) is an autoimmune disease characterized by vasculopathy, immune dysregulation, and multi-organ fibrosis. Interstitial lung disease (ILD) is a complication of SSc and a leading cause of SSc-death. The administration of hypochlorous acid (HOCl) intradermally in the mouse (HOCl-SSc) purportedly shows several features typical of SSc. We studied the model by injecting BALB/c mice daily intradermally with HOCl for 6-weeks, an exposure reported to induce lung fibrosis. On day 42, the skinfold thickness and the dermal thickness were two and three times larger respectively in the HOCl group compared to controls. HOCl treatment did not result in histological features of pulmonary fibrosis nor significant changes in lung compliance. Automated image analysis of HOCl mice lungs stained with picrosirius red did not show increased collagen deposition. HOCl injections did not increase pulmonary mRNA expression of pro-fibrotic genes nor induced the production of serum advanced oxidation protein products and anti-topoisomerase 1 antibodies. Immune cells in bronchoalveolar lavage fluid (BALF) and whole lung digests were not increased in HOCl-treated animals. Since lung fibrosis is proposed to be triggered by oxidative stress, we injected HOCl to Nrf2^−/−^ mice, a mouse deficient in many antioxidant proteins. Lung compliance, histology, and BALF leukocyte numbers were comparable between Nrf2^−/−^ mice and wild-type controls. We conclude that the HOCl-SSc model does not manifest SSc-lung disease.

## Introduction

Systemic Sclerosis (SSc), or scleroderma, is a rare autoimmune disease characterized by the distinctive pathological triad of vasculopathy, immune dysregulation, and fibrosis of the skin and internal organs^1^. SSc carries the highest case-based mortality and morbidity among the rheumatic diseases and places a significant burden on patients^1,2^. Skin thickening and Raynaud’s phenomenon are key SSc clinical features whereas progressive interstitial lung disease (ILD) accounts for 18% of all SSc-deaths^3^. Although the mechanisms underlying the pathogenesis of SSc remain elusive, there is evidence of endothelial cell activation resulting in vascular damage, disruption of immune tolerance with the presence of autoantibodies such as anti-topoisomerase 1 (anti-Scl-70), and overproduction of profibrotic mediators^1^. Mechanistically, oxidative stress is considered central to SSc pathogenesis^4^.

Fibrosis, the distinguishing hallmark of SSc, affects skin and visceral organs and can lead to organ failure^1^. Fibrosis is characterized by the replacement of normal tissue with mechanically stressed, rigid extracellular matrix components such as collagen and fibronectin resulting in altered tissue architecture^5^. Interstitial lung disease (ILD) comprises over 200 different lung disorders characterized by varying degrees of inflammation and fibrosis of the lung parenchyma^6^. ILD patients clinically manifest with progressive dyspnea, cough, and reduced exercise tolerance. Notably, ILD is a frequent complication of SSc. Nintedanib, an intracellular inhibitor of tyrosine kinases^7^, and tocilizumab, an IL-6 inhibitor, are currently the only medications approved by the US Food and Drug Administration for the treatment of SSc-ILD^8^. These therapies slow the rate of decline of the forced vital capacity but do not reverse established lung fibrosis. Therefore, there is a need for therapies that promote fibrosis resolution and for pertinent ILD pre-clinical models.

Among SSc-ILD models, the hypochlorous acid mouse model (HOCl-SSc) is favoured as it is proposed to recapitulate key features of SSc including anti-Scl-70 antibody production, skin lesions reminiscent of SSc and concomitant lung fibrosis^4,9–13^. Daily intradermal HOCl injections are reported to induce inflammation in the dermis^11^, histological skin fibrosis^4,11,12,14,15^, increase skinfold and dermal thickness^4,11,12,14,15^, increase skin collagen content^11,12^, and increase mRNA levels of collagen genes *Col1a1*, *Col3a1*, transforming growth factor*-*β (*TGF*-*β*), and α-smooth muscle specific actin (*ACTA2*)^12^. With respect to the lungs, an increase in lung collagen content^4,9,12,13,16^ and an increase in lung mRNA and protein levels of profibrotic mediators such as α-SMA^4,12^, Col1a1^4,12^, Col3a1^12^, and TGF-β^12^, are also reported^4,9,10,12,16,17^. Kidney fibrosis^9^ and intimal vascular abnormalities are described in the HOCl-SSc model^9,17^.

Mechanistically, HOCl injected intradermally is proposed to generate lung fibrosis through excess formation of reactive oxygen species (ROS) and oxidative stress induction. Specifically, advanced oxidized serum proteins (i.e. AOPPs) were suggested to mediate the propagation of oxidative stress from the skin to the lungs^9^. There are several reports of increased AOPP levels in HOCl-treated mice^9,12,13^. Nrf2 is a redox-sensitive transcription factor that regulates cellular responses against oxidative stress^18^. Intradermal HOCl injections administered to Nrf2^−/−^ mice are described to lead to increased skin thickness, collagen content, and more severe skin pathology than in wild type animals similarly treated^4^. Likewise, increased lung collagen content and exacerbated lung pathology were reported in Nrf2^−/−^ HOCl-injected mice^4^. However, lung function tests, quantitative histopathological image analysis, and in-depth characterization of lung immune cell phenotypes have not been conducted neither in BALB/c nor in Nrf2^−/−^ mice. In the current study, we characterized the effect of chronic HOCl administration on skin and lung histology and respiratory mechanics in BALB/c, C57BL/6 and Nrf2^−/−^ mice. The findings of the HOCl murine model were contrasted to a well-characterized model of pulmonary fibrosis, namely the subcutaneous administration of bleomycin via osmotic minipump [(BLM-MP) model]^19^.

## Methods

### Animals

The ‘Animal Research: Reporting of In Vivo Experiments’ (ARRIVE) guidelines were used^20^. For the HOCl-SSc mouse model, female BALB/c mice (6–8-week-old) were purchased from Charles River Laboratories (St. Constant, QC, Canada), while Nrf2^−/−^ mice on a C57BL/6 background and corresponding wild type mice were purchased from Jackson Laboratories (Bar Harbor, Maine, United States). For the BLM mouse model, male C57Bl/6, 9–10-week-old mice were purchased from Jackson Laboratories. Animals were housed in a conventional animal facility at the Research Institute of the McGill University Health Centre (RI-MUHC). Animals were treated in accordance with the guidelines of the Canadian Council of Animal Care (CCAC) and protocols were approved by the Animal Care Committee of McGill University.

### HOCl-SSc model: experimental design and preparation of hypochlorous acid (HOCl) solution

To induce lung fibrosis, we followed reported methods^4,9–12,15^. Mice were anesthetized with isoflurane (4% induction, 1.5–2.5% maintenance) and received intradermal injections of HOCl in two sites of the lower back (150 µL in each site, a total of 300 µL) five days a week for 6 weeks. After 6 weeks, injections were discontinued, and experimental readouts were assessed^9,11^. Additional experiments were conducted to test the effects of increasing the total volume of the injected solution to 400 µL (200 µL per site) and prolonging the duration of injections for 2 additional weeks (8 weeks total). Different brands of commercially available bleach were also tested.

Potassium dihydrogen phosphate (KH_2_PO_4_) buffer solution was prepared at a concentration of 100 mM with a pH of 6.2, as previously established^11^. The KH_2_PO_4_ solution was stored at 4 °C for a maximum of 3 months. Commercial bleach at a concentration of 4% was used. The bleach bottle was replaced every 3 weeks, once opened. The HOCl solution was prepared at a final concentration of 0.096% by addition of the buffer solution. The optical density was measured at a wavelength of 292 nm by spectrophotometry and was between 0.7 and 0.9 (arbitrary units). The HOCl solution was prepared fresh daily. The control group received injections of an equal volume of PBS^9,11^.

### Bleomycin-minipump (BLM-MP) mouse model protocol

Osmotic minipumps (ALZET 1007D; DURECT, CA) containing 100 µL of BLM (60 mg/kg) (Adooq. Bioscience, A10152-10) or phosphate buffer saline (PBS) were implanted subcutaneously in ten-week-old C57BL/6 mice as previously described^19^. Minipump contents were delivered as a continuous infusion at a rate of 0.5 µL per hour for 7 days^19^. Pumps were removed on day 10 as per manufacturer’s recommendations. Mice were sacrificed on day 28 and readouts were assessed.

### Skin thickness

Skin thickness was measured with a digital caliper (Mitutoyo 547-500S) at three different locations on the lower back of the mouse near the site of injections. The average of the 3 measurements was recorded.

### Measurement of respiratory mechanics

To evaluate lung function, we followed methods previously described^21,22^. Mice were sedated with xylazine (8 mg/kg; intraperitoneally) and anesthetized with pentobarbital sodium (30 mg/kg; intraperitoneally). They were tracheostomized and an 18-gauge metal cannula was inserted and connected to a computer-controlled small animal ventilator (flexiVent™, SCIREQ, Montreal, PQ, Canada). Mice were mechanically ventilated at 150 breaths/minute with a positive end expiratory pressure (PEEP) of 3cmH_2_O. Next, a muscle relaxant was administered (rocuronium bromide; 2 mg/kg) intraperitoneally. This was followed by 3 min of mechanical ventilation to allow the mice to stabilize. Respiratory mechanical properties were assessed using the commercial software referred to as the *mouse mechanics scan*^23^.

### Pressure volume (PV) curve

The static P–V relationship was used to assess the static compliance of the lung. It was constructed by a stepwise inflation of the respiratory system from a positive end expiratory pressure (PEEP) of 3cmH_2_O to 30 cmH_2_O and a slow deflation in a similar manner^23^. This procedure was repeated three times and data were averaged to generate a single P–V curve. During the inflation and deflation, transrespiratory pressure and volume were recorded. The static compliance was obtained from the slope of a line segment connecting two points within the linear range of the expiratory limb.

### Bronchoalveolar lavage (BAL) fluid analysis

BAL fluid processing and analysis were performed as previously published^22^. Following lung mechanics measurements, BAL was performed by instilling 1 mL of cold PBS via the tracheal cannula, with a subsequent recovery of approximately 0.8 mL of fluid by gentle suction. BAL fluid was centrifuged at 3000 rpm for 5 min. The cell pellet was resuspended in PBS and cells were counted on a haemacytometer. The BAL cell suspension was deposited on a glass slide by cytocentrifugation at 1000 rpm for 5 min (Epredia™ Cytospin™ 4 Cytocentrifuge, Shandon, Pittsburgh, PA, USA). Cells were fixed and stained with Diff-Quik (Fisher Scientific, Kalamazoo, MI). Differential cell counts were determined by calculating the percent of macrophages, lymphocytes, neutrophils, and epithelial cells from a count of three hundred cells.

### Tissue collection and histochemical staining

On day 42, skin biopsies from the dorsum of the animals within the area of injections were performed. Following harvesting, lungs were inflated through the tracheal cannula with 10% buffered formalin with a distending pressure of 25cmH_2_O for 10 min^24^. The trachea was then tied off with a thread and the inflated lungs were removed. The skin and lungs were both submerged in 10% formalin for 24–48 h prior to processing and embedding in paraffin^24^. Tissues were cut into 4 µm sections and stained with hematoxylin and eosin (H&E), Masson’s Trichome (MT) (Sigma-Aldrich, St. Louis, Missouri), and picrosirius red (PSR) (Abcam, ab150681) according to the manufacturer’s instructions.

### Picrosirius red staining and analysis

Skin and lung sections were deparaffinized, hydrated, stained with PSR, and scanned using the Aperio® AT Turbo Scanner (Leica Biosystems, Concord, ON, Canada). Automated image analysis was performed on 3 non-contiguous sections from the left lung using the Aperio Pixel Count Algorithm in the Aperio ImageScope software (Leica Microsystems, Richmond hill, ON, Canada). The algorithm detects pixels that match the input parameters which are based on the hue, saturation, and intensity color model. To detect collagen with PSR, the default hue value was used (0.10), a hue width of 0.40 and color saturation of 0.08 were specified^25,26^. Collagen content (expressed in pixels) was corrected to the area of each lung determined by manual digitization of the outer perimeter of the lung.

### Quantification of dermal thickness

Masson’s Trichrome or PSR stained slides of mouse skin sections were scanned using the Aperio Scanner and analyzed using the Aperio ImageScope software at 20 × magnification. Dermal thickness was measured from the dermal–epidermal junction to the dermal-adipose junction. Either two or three skin biopsies per mouse were used for analysis. Three random measurements were taken per section.

### Advanced oxidation protein products (AOPPs)

The protocol used for obtaining serum was adapted from “Blood Collection and Sample Preparation for Rodents” (IDEX BioAnalytics)^27^. Blood from PBS and HOCl-injected mice was obtained after the completion of FlexiVent measurement by cardiac puncture and collected in tubes (BD Microtainer®). Tubes were centrifuged at 3800 rpm for 10 min (Eppendorf® centrifuge 5424). The serum was aliquoted in 1.5 mL Eppendorf tubes and stored at − 20 °C until the levels of AOPPs and anti-Scl-70 antibodies were determined.

Serum from PBS and HOCl-injected mice was diluted 1/20 in PBS and 200 μL was pipetted in a 96-well plate. A solution of chloramine T (100 μM) was used to prepare the standard curve. All samples and standards were run in duplicates. An aliquot of 10 μL of 1.16 M potassium iodide was added to each well, mixed and incubated for 5 min at room temperature. Next, 20 μL of acetic acid solution was added to each well to stop the reaction. Optical density was read at 340 nm on a microplate spectrophotometer (LKB Pharmacia 4050 UltroSpec II UV–Vis). AOPP content was calculated using the chloramine T standard curve^9^.

### Anti-topoisomerase I autoantibodies (anti-Scl-70)

A mouse anti-Scl-70 ELISA kit (Signosis, EA-5205) was used. The ELISA was conducted following the specifications of the manufacturer. The kit contained a positive and negative control. Optical density was read at 540 nm on a microplate spectrophotometer.

### Lung hydroxyproline assay

Lung hydroxyproline content was analyzed with a hydroxyproline colorimetric assay kit (Abcam, Ab222941) following manufacturer’s instruction. The lungs from PBS and HOCl mice were hydrolyzed in 10N NaOH for 1 h at 120 °C to obtain individual amino acids. Cold samples were neutralized with 10 N HCl. Hydroxyproline was converted to pyrolle-2-carboxylate by oxidation via addition of chloramine-T. 3-dimethylamino benzoic acid was added to the intermediate product and incubated at 65 °C for 45 min forming a colored complex. The absorbance was measured at 560 nm using a microplate reader (LKB Pharmacia 4050 UltroSpec II UV–Vis) and expressed as micrograms of hydroxyproline per milligram of lung^28^.

### RNA isolation

The mouse right lung and an amount of skin of approximately 30 mg were dissected, transferred to 2 mL screw-top tubes (Heathrow Scientific HEA10060), snap frozen and stored at − 80 °C until the RNA isolation. Homogenization beads and lysis buffer with tris(2-carboxyethyl) phosphine (TCEP) was added to the frozen tissues and homogenized (607, Mini-BeadBeater-16). Lung and skin RNA were extracted using the Nucleospin® RNA kit (Macherey–Nagel™ 740955.50) and RNeasy Mini Kit (Qiagen, Inc), respectively, according to the manufacturer’s instructions. The purity of RNA was verified by a microplate reader (Take3™ Micro-Volume Plate) using the Gen 5™ software.

### Reverse transcription and real-time quantitative PCR

Following RNA isolation, cDNA was synthesized with the LunaScript® RT SuperMix Kit (NEB #E3010) according to the manufacturer’s instructions using a thermocycler (Applied Biosystems™ Veriti™). A total of 500 ng total RNA was reverse transcribed into cDNA. Real time quantitative PCR was performed using SYBR® Green PCR Master Mix (Applied Biosystems, Foster City, CA). Primer sequences were used to determine alpha-smooth muscle actin (*ACTA2*), transforming growth factor beta 1 (*TGF-β1*), fibronectin (*FN*), collagen type IV alpha 1 (*Col4A1*), Collagen type I alpha 1 (*Col1A1*), collagen type III alpha I (*Col3A1*), peptidylpropyl isomerase A (*PPIA*). *ACTA2* forward; GAGGCACCACTGAACCCTAA, reverse; ATCTCCAGAGTCCAGCACA. *TGF-β1* forward; ACGTCACTGGAGTTGTACGG, reverse; TGGGGCTGATCCCGTTGA. *FN* forward; CGAGGTGACAGAGACCACAA, reverse; CTGGAGTCAAGCCAGACACA. *Col4A1* forward; AGGGTTACCTGGAGAAAAAGGG, reverse; TGGTCTCCTTTCTGTCCCTTC. *Col1A1* forward; ACCTTCCTGCGCCTAATGTC, reverse; AGTTCCGGTGTGACTCGTG. *Col3A1* forward; TCCCCTGGAATCTGTGAATC, reverse; TGAGTCGAATTGGGGAGAAT. *PPIA* forward; CTGTAGCTCAGGAGAGCGTC, reverse; CCAGCTAGACTTGAAGGGGAA. cDNA was amplified using Step-One-Plus machine (Applied Biosystems). Relative mRNA expression was calculated using the ΔΔCt method. Data were normalized to the housekeeping gene, Peptidylprolyl Isomerase A (*PPIA*).

### Lung immune cell assessment

Both mouse lungs were removed and were placed on ice in a 15 mL tube containing 2 mL of RPMI-1640 medium. Lungs were transferred into a 6-well plate containing collagenase (Sigma-Aldrich, C5138) at a final concentration of 150 U/mL. Lungs were minced and incubated for 45 min at 37 °C and 5% CO_2_/balance air. The minced lung was passed through a 70 μm nylon strainer into a 50 mL tube. The cell suspension was centrifuged at 1700 rpm for 10 min. Red blood cells (RBC) were then lysed using the RBC lysis buffer (BioLegend, 420301). The cell suspension was centrifuged at 1700 rpm for 7 min and re-suspended in 10 mL of RPMI media. Cells were counted on a hemacytometer.

For immunophenotyping of leukocyte subsets, 1.5 × 10^6^ cells from each lung sample were plated in 96-well v-bottom plates. Cells were stained with eFluor506 viability dye (1:1000 dilution, eBioscience™) and incubated at 4 °C. Anti-CD32/16 was used to block Fc receptors (BioLegend, B266361). Cells were stained with the following antibodies: rat anti-mouse monoclonal Siglec-F-BV786 (BD Biosciences, Cat. No. 740956), hamster anti-mouse monoclonal CD11b-BUV395 (BD Biosciences, Cat. No. 563553), hamster anti-mouse CD11c-PeCy7 monoclonal (BD Biosciences, Cat. No. 561022), rat anti-mouse monoclonal F4/80-APC (BioLegend®, Cat. No. 123116), rat anti-mouse monoclonal Ly6C-FITC (BioLegend®, Cat. No. 108405), rat anti-mouse monoclonal Ly6G-Percp eflour 710 (BioLegend®, Cat. No. 46-9668-82).

Cells were fixed using the fixation reagent and diluent (Invitrogen™). All samples were acquired using the LSRFortessa x-20 flow cytometer. Analysis was performed using FlowJo V10 software. Fluorescence minus one (FMO) controls were used to set the upper boundary for background signal on the omitted label, and to identify and gate positive populations. The gating strategy is outlined in Supplementary Fig. 1.

### Statistical analysis

GraphPad Prism 9 software (GraphPad Software Inc., San Diego, CA, USA) was used for statistical analysis. Data are expressed as mean ± standard error of the mean (SEM). Non-parametric Mann–Whitney *U* test was used to compare two groups. Two-way ANOVA was used for analysis of P–V loops. A *p*-value less that 0.05 was considered statistically significant.

## Results

### HOCl injections induce local skin fibrosis whereas BLM-MPs induce distal skin fibrosis

**Figure 1:**
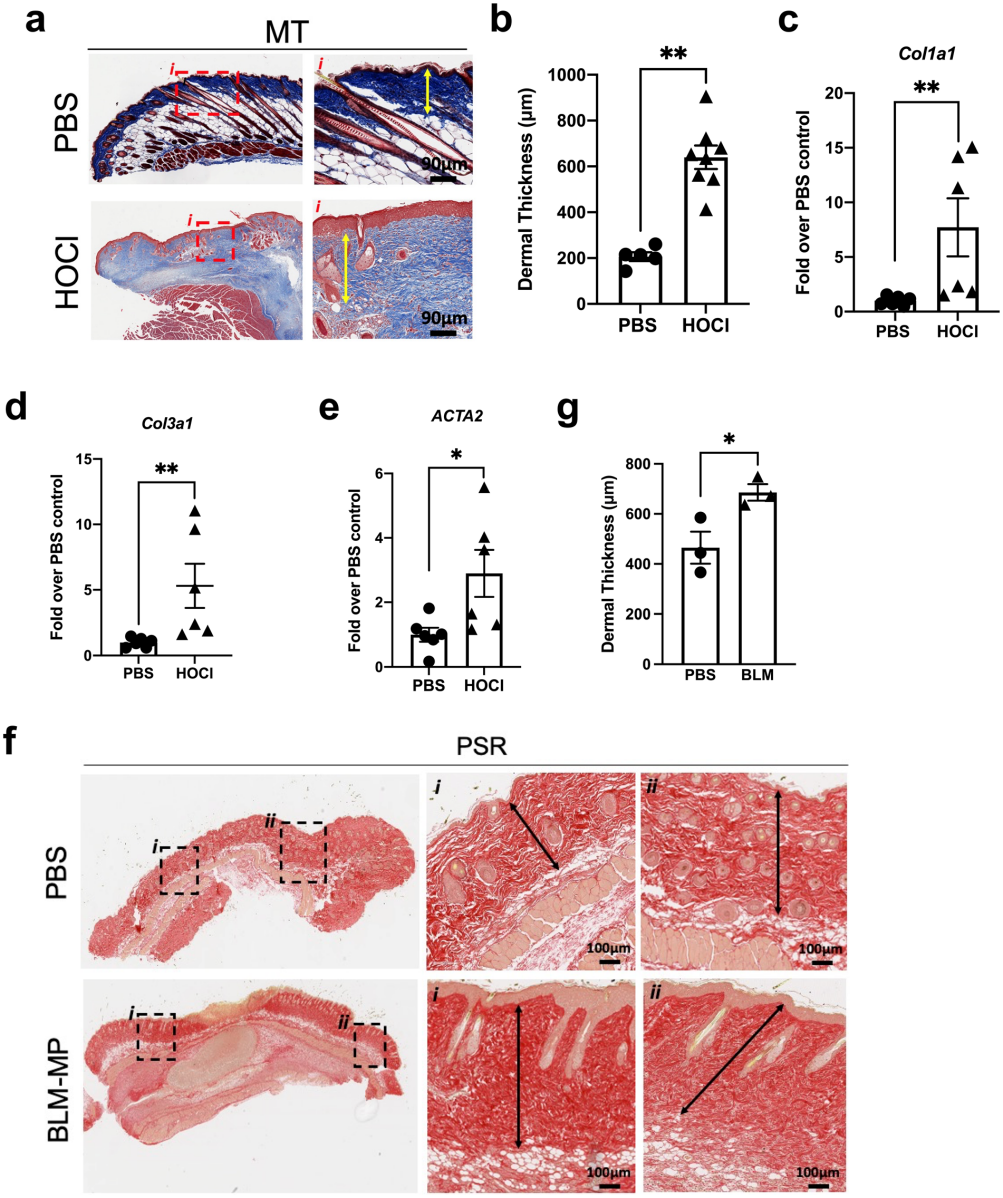
HOCl injections induce local skin fibrosis whereas BLM administration induces skin fibrosis distal to minipump implantation. (**a**) Representative skin image of MT staining of PBS and HOCl-treated mice. Biopsies were obtained from the site of injection. Low magnification and × 20 magnification. Scale bar: 90 μm. Yellow arrows show dermal measurements. (**b**) Average measurements of dermal thickness.* n* = 5–8 mice per group, **p = 1.60** × **10^–3^. (**c**) mRNA expression of *Col1a1* (**c**), *Col3a1* (**d**), and *ACTA2* (**e**) in PBS and HOCl mice. *n* = 6 mice per group, **p < 5.0** × **10^–3^. (**f**) Representative skin images of PSR staining of PBS and BLM-MP mice. Low magnification and × 20 magnification. Scale bar: 100 μm. Black arrows show dermal thickness measurements (**g**). Average measurements of dermal thickness n = 3 mice per group, *p = 3.75 × 10^–2^. Data are represented as mean ± SEM from 2–3 independent pooled experiments (**a**–**e**). Data were analyzed using Mann–Whitney *U* test (**b**–**e**, **g**).

To assess skin fibrosis in HOCl-injected mice, we measured skinfold thickness with calipers, performed histological assessment of H&E and MT-stained sections, and evaluated mRNA expression of profibrotic genes. Compared to PBS-injected mice, HOCl mice had increased skin thickness at all timepoints (Supplementary Fig. 1a). H&E-stained skin sections of HOCl-injected mice showed areas of epidermal hyperplasia, hyperkeratosis, and loss of adipose tissue and appendages. Those affected areas alternated with less or unaffected areas (Supplementary Fig. 1b). Inflammation extending into the superficial layer of the muscle bundles was also observed (Supplementary Fig. 1b). MT sections of HOCl-injected mice showed both areas of irregular and homogeneous collagen bundles in the dermis. Evidence of fibrosis was also observed in the deep reticular dermis (Fig. 1a). Histological assessment showed both HOCl and BLM-MP mice to have increased dermal thickness compared to PBS mice (Fig. 1b,g). However, HOCl skin thickness was only increased at the site of injections whereas BLM-MP mice had increased dermal thickness at a distance from the site of minipump implantation. Lastly, mRNA expression of *Col1a1*, *Col3a1,* and *ACTA2* was elevated in HOCl treated mice (Fig. 1c–e).

### HOCl injections neither induce lung histologic abnormalities nor impair lung function

**Figure 2:**
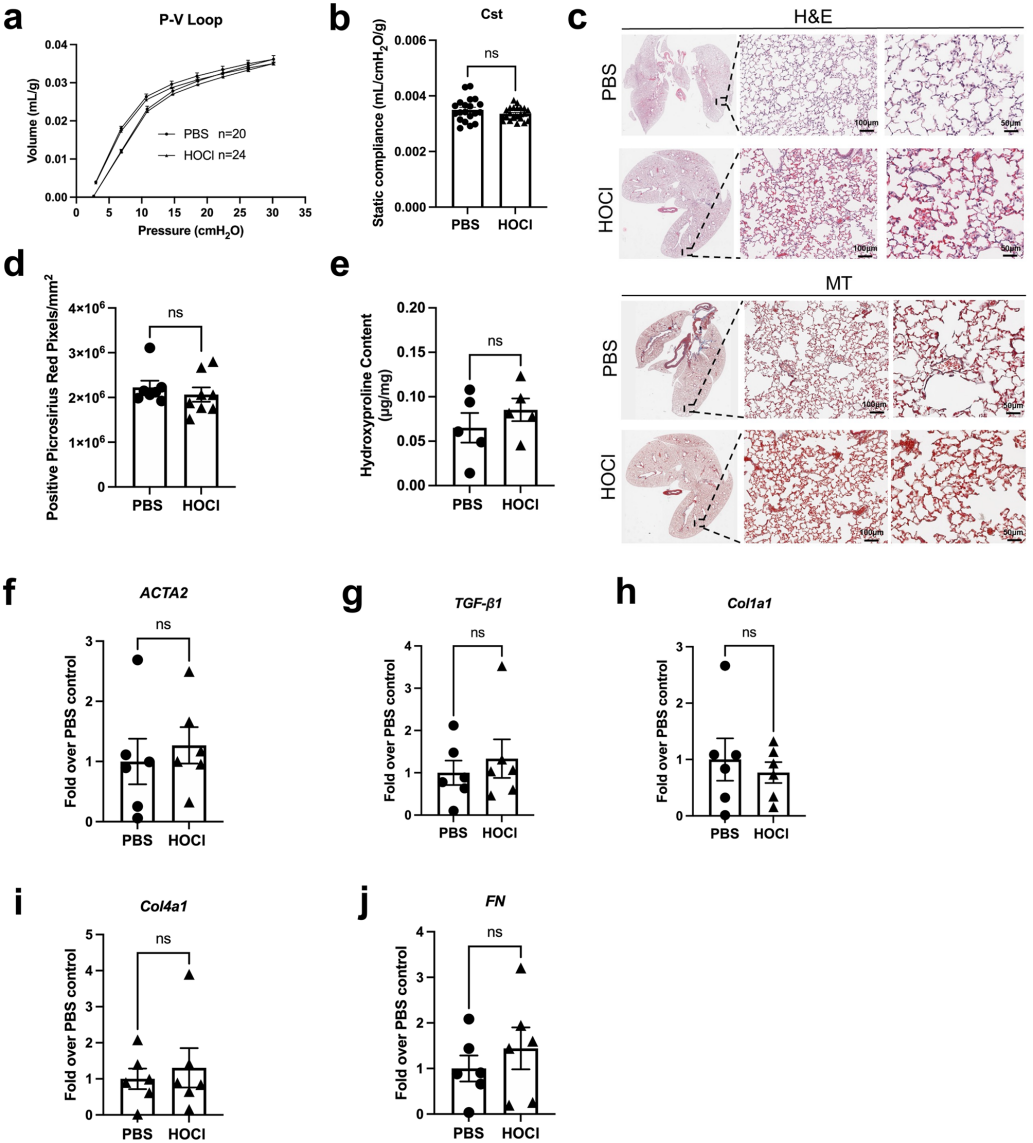
Intradermal HOCl injections (6 weeks) did not impair lung function or induce lung fibrosis. (**a**) Mean pressure–volume (P–V) loops for PBS and HOCl-injected mice. (**b**) Static lung compliance (C_st_) (n = 20–24). (**c**) Micrographs of H&E and MT—stained lung sections (low magnification and × 20 and × 40 magnification) (n = 11–15). Scale bar: 100 μm (× 20 magnification) and 50 μm (× 40 magnification). (**d**) Image analysis of PSR-stained slides (n = 7–8). (**e**) lung hydroxyproline content in PBS and HOCl-injected animals (n = 6). (**f**) relative gene expression of α-smooth muscle actin (*ACTA2*), (**g**) transforming growth factor β1 (*TGF- β1*), (**h**) collagen type I alpha 1 (*Col1a1*), (**i**) collagen type IV alpha 1 (*Col4a1*), and (**j**) fibronectin (*FN*) in lung tissue. Data are represented as mean ± SEM from 2 to 4 independent pooled experiments. Data were analyzed using Two-way ANOVA (**a**) and Mann–Whitney *U* test (**b**, **d**–**j**). *ns* not significant.

We determined the effect of HOCl on lung compliance by performing in vivo lung function measurements (flexiVent). At Day 42, there was no difference between the P–V loops of PBS and HOCl-injected mice (Fig. 2a). Static compliance confirmed the lack of difference between the two groups (PBS vs HOCl: 3.50 × 10^–3^ ± 9.30 × 10^–5^ vs 3.36 × 10^–3^ ± 4.61 × 10^–5^ mL/cmH_2_O/g) (Fig. 2b). H&E and MT-stained lung tissue sections showed no histological abnormalities (Fig. 2c). Quantitative image analysis performed on PSR-stained slides showed no differences in the number of positive pixels between PBS and HOCl treated mice (PBS vs HOCl: 2.22 × 10^6^ ± 1.52 × 10^5^ vs 2.06 × 10^6^ ± 1.60 × 10^5^ positive PSR pixels/mm^2^) (Fig. 2d). The collagen content in the lungs of HOCl mice was not increased (Fig. 2e). Furthermore, no difference was observed in the mRNA expression of pro-fibrotic markers *ACTA2*, *TGF-β1*, *Col1a1*, *Col4a1* and *FN* (Fig. 2f–j). In an attempt to elicit lung fibrosis, we prolonged the duration of HOCl injections. Administration of HOCl for a period of 8 weeks had no effect on lung compliance (PBS vs HOCl: 3.19 × 10^–3^ ± 4.25 × 10^–4^ vs 2.82 × 10^–3^ ± 2.15 × 10^–4^ mL/cmH_2_O/g) (Supplementary Fig. 2b). H&E-stained sections from HOCl injected mice were comparable to those of PBS injected mice (Supplementary Fig. 2c). MT-stained sections did not show any evidence of increased collagen deposition in the lung parenchyma (Supplementary Fig. 2d). Together, these data indicate that chronic exposure to intradermal HOCl injections does not result in lung fibrosis.

### BLM-MPs generates abnormalities in pulmonary histology and impairs lung function

**Figure 3:**
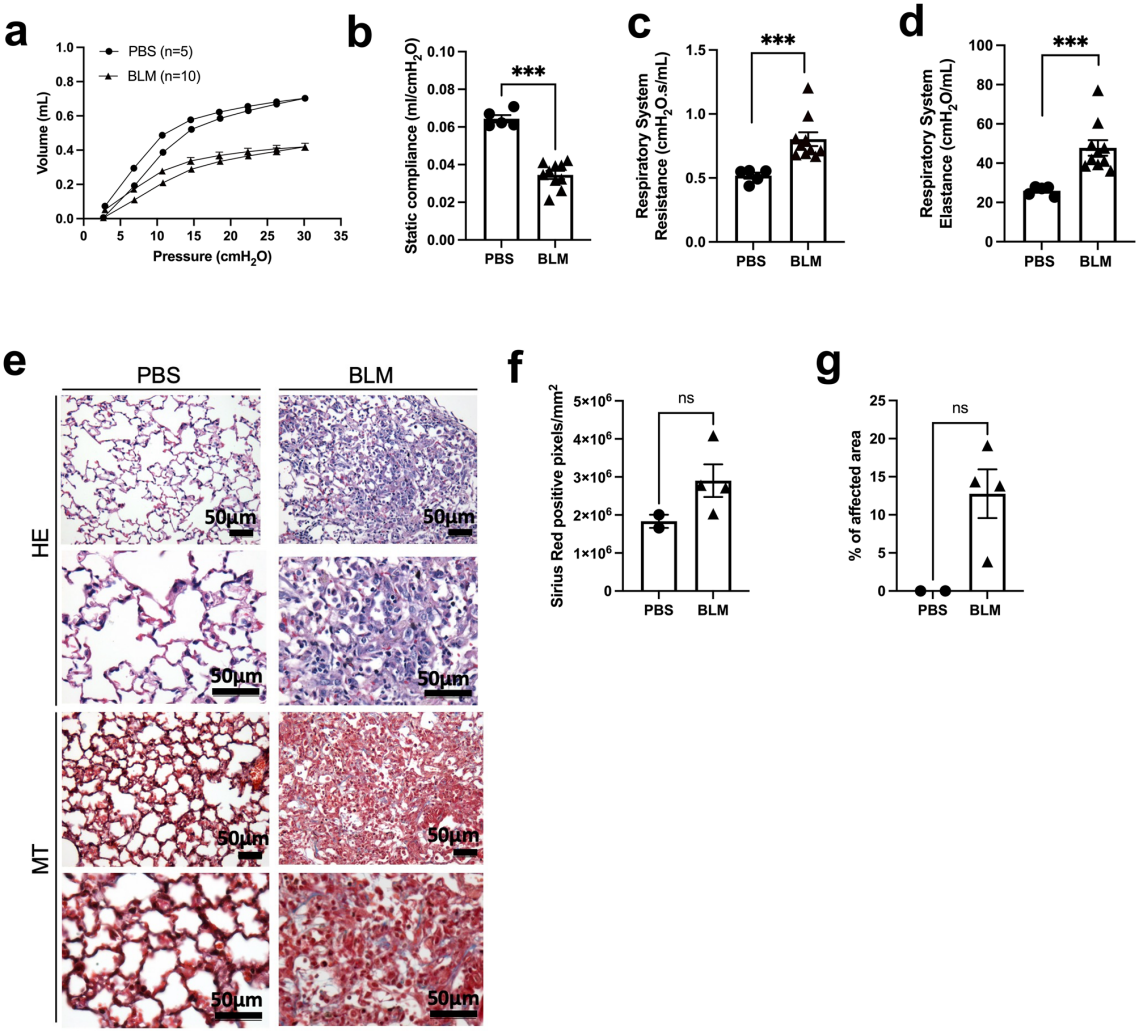
At day 28 the BLM minipump model leads to impaired lung mechanics with interstitial inflammation and fibroblastic foci. (**a**) Mean pressure–volume (P–V) loops for PBS and BLM-treated mice. (**b**) Static lung compliance (C_st_). (**c**) Lung resistance (Rrs) and elastance (Ers) (**d**) calculated by the flexiVent software by fitting the data from the single frequency forced oscillation manoeuvre to the single-compartment model. *n* = 5–10 mice per group, graphs show mean ± SEM. Two tailed t test, *p < 0.005 (**e**). Hematoxylin and eosin (HE) and Masson’s Trichrome (MT)-stained lung sections. First row of HE and MT × 20 images and bottom row × 40 magnification. Scale bar: 50 μm. (**f**) Image analysis on PSR-stained slides and (**g**). Abnormal lung areas quantified by the Positive Pixel Count V9 Aperio Algorithm (airways and large vessels were excluded). Three transverse sections per left lung were analyzed (lower, middle, upper lobe). Mann–Whitney *U* Test was used for comparisons. *ns* not significant.

The degree of lung injury induced by bleomycin was evaluated by histology, pulmonary function tests and micro-CT imaging. Four weeks post-pump implantation, BLM-MP mice had decreased lung compliance, increased resistance, and dynamic elastance compared to control animals (Fig. 3a–d). H&E-stained lung sections showed a predominantly inflammatory process with minimal evidence of fibrosis. The distribution of parenchymal injury was largely peripheral which resembles the pattern in interstitial lung disease (Fig. 3e). In the histology quantification, BLM-MP animals tend to have higher number of positive picrosirius red pixels and affected lung area (Fig. 3f–g). The presence of increased areas of attenuation on micro-CT corroborated the BLM-induced lung injury (Supplementary Fig. 3).

### Inflammatory infiltrates in BALF and collagenase digests of lung tissue are similar in PBS and HOCl-injected mice

**Figure 4:**
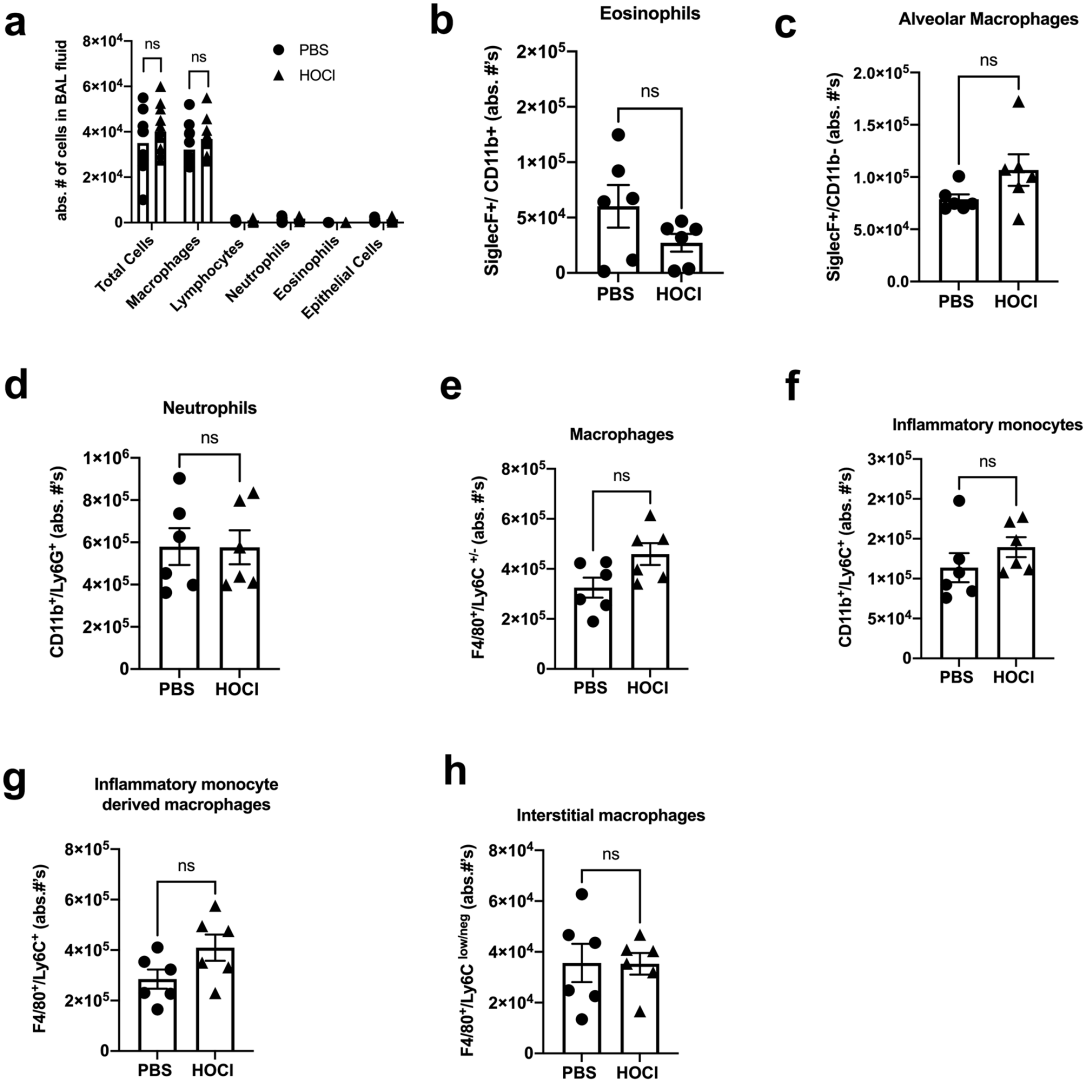
HOCl injections do not induce lung inflammation. (**a**) Total and differential cell count from BALF of HOCl and PBS -injected mice (n = 9–15). Quantification of total numbers of (**b**) eosinophils (SiglecF^+^, CD11b^+^), (**c**) alveolar macrophages (SiglecF^+^, CD11b^−^), (**d**) neutrophils (SiglecF^−^, CD11b^+^, Ly6G^+^), (**e**) macrophages (SiglecF^−^,CD11b^+^, Ly6G^−^, F4/80^+^, Ly6C^+/−^), (**f**) inflammatory monocytes (SiglecF^−^, CD11b^+^, Ly6G^−^, F4/80^−^, Ly6C^+^), (**g**) inflammatory monocyte-derived macrophages (SiglecF^−^, CD11b^+^, F4/80^+^, Ly6C^+^), and (**h**) interstitial macrophages (SiglecF^−^, CD11b^+^, F4/80^+^, Ly6C^low/neg^) (n = 6). Data are represented as mean ± SEM from 2 to 4 experiments Data were analyzed using Two-way ANOVA (**a**) and Mann–Whitney *U* test (**b**–**h**). *ns* not significant.

Inflammatory cells are reported to precede and mediate the development of fibrosis by releasing a variety of proinflammatory factors that inflict tissue injury^29–32^. Therefore, we tested whether intradermal HOCl injections induced lung inflammation. Evaluation of BALF cells showed no difference in total white blood cell counts and differential cell counts between PBS and HOCl-injected animals (Fig. 4a). Lung tissue digests were analyzed by flow cytometry in a separate group of mice. Representative contour plots of the flow cytometry gating strategy are shown in Supplementary Fig. 4. Compared to PBS mice, HOCl injected mice showed no differences in the number of eosinophils (PBS vs HOCl, absolute numbers per lung: 60,213 ± 19,221 vs 27,265 ± 7991), alveolar macrophages (PBS vs HOCl, absolute numbers: 45065 ± 7205 vs 63,792 ± 10,779), neutrophils (PBS vs HOCl, absolute numbers: 579,732 ± 87,053 vs 576,460 ± 80,495), macrophages (PBS vs HOCl, absolute numbers: 324,930 ± 40,016 vs 459,118 ± 43,661), inflammatory monocytes (PBS vs HOCl, absolute numbers: 113,795 ± 18,204 vs 139,415 ± 12,532), inflammatory monocyte derived macrophages (PBS vs HOCl, absolute numbers: 284,930 ± 37,756 vs 409,492 ± 52,010) and interstitial macrophages (PBS vs HOCl, absolute numbers: 35,623 ± 7524 vs 35,310 ± 4262) (Fig. 4b–h). These findings indicate a lack of an inflammatory response following HOCl treatment.

### HOCl injections do not induce AOPPs or anti-Scl-70 antibodies production

**Figure 5:**
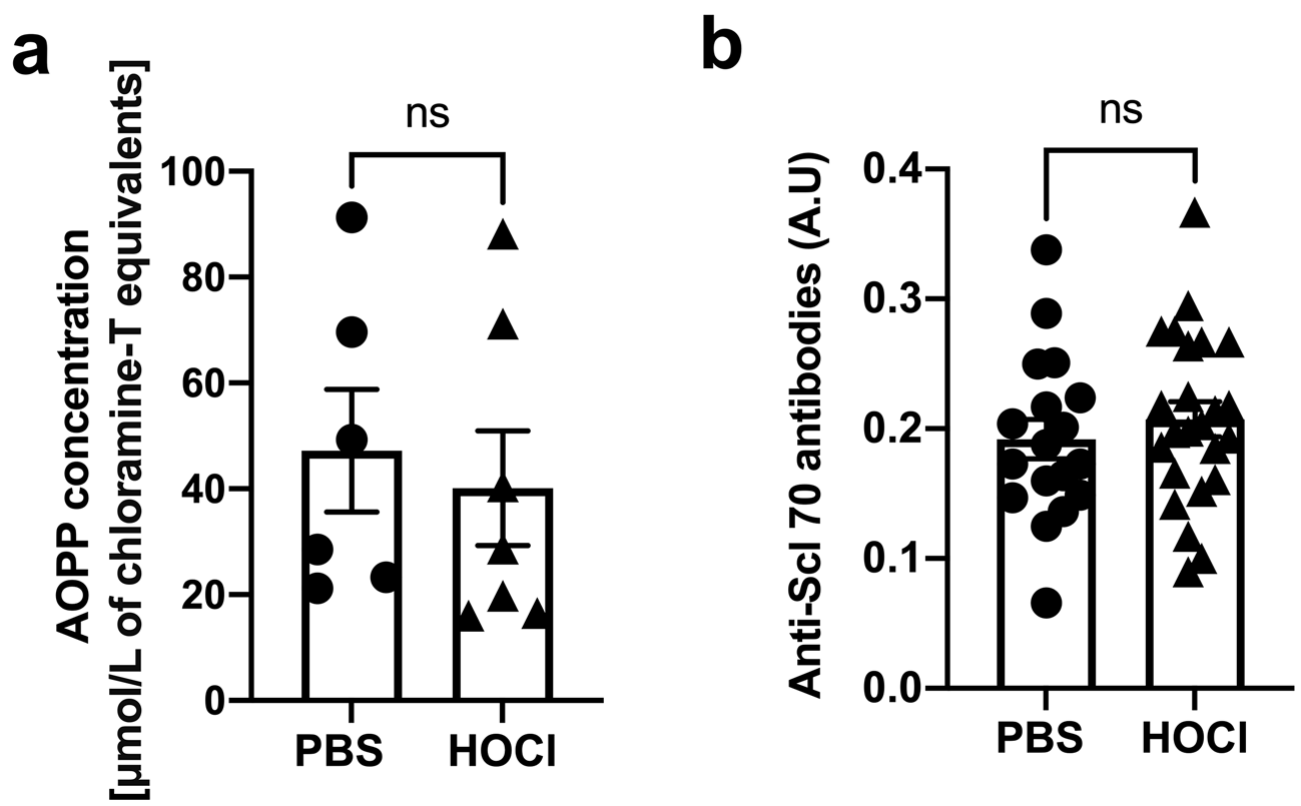
Intradermal HOCl injections neither induced the production of AOPPs nor anti-Scl-70 antibodies. (**a**) AOPPs concentration in sera from PBS and HOCl-injected animals (*n* = 6)*.* (**b**) anti-Scl-70 serum levels (*n* = 18–25). Data are represented as mean ± SEM from 2–6 experiments. Data were analyzed using Mann–Whitney *U* test (**a**, **b**). Abbreviations: ns: not significant.

HOCl purportedly leads to lung fibrosis by inducing oxidative stress in the skin that leads to AOPPs that propagate the injury to the lungs^9^. However, we did not observe increased concentrations of AOPPs in the sera of HOCl mice (PBS vs HOCl: 47.21 ± 11.62 vs 40.12 ± 10.87 µmol/L) (Fig. 5a). The development of fibrosis was also reported to be associated with the production of anti-Scl-70 antibodies^9^. HOCl mice were seronegative for anti-Scl-70 (PBS vs HOCl, arbitrary units: 0.19 ± 0.02 vs 0.21 ± 0.01) (Fig. 5b). These data fail to demonstrate systemic involvement following HOCl injections.

### HOCl injections induced comparable skin fibrosis in Nrf2^−/−^ mice and wild-type controls

**Figure 6:**
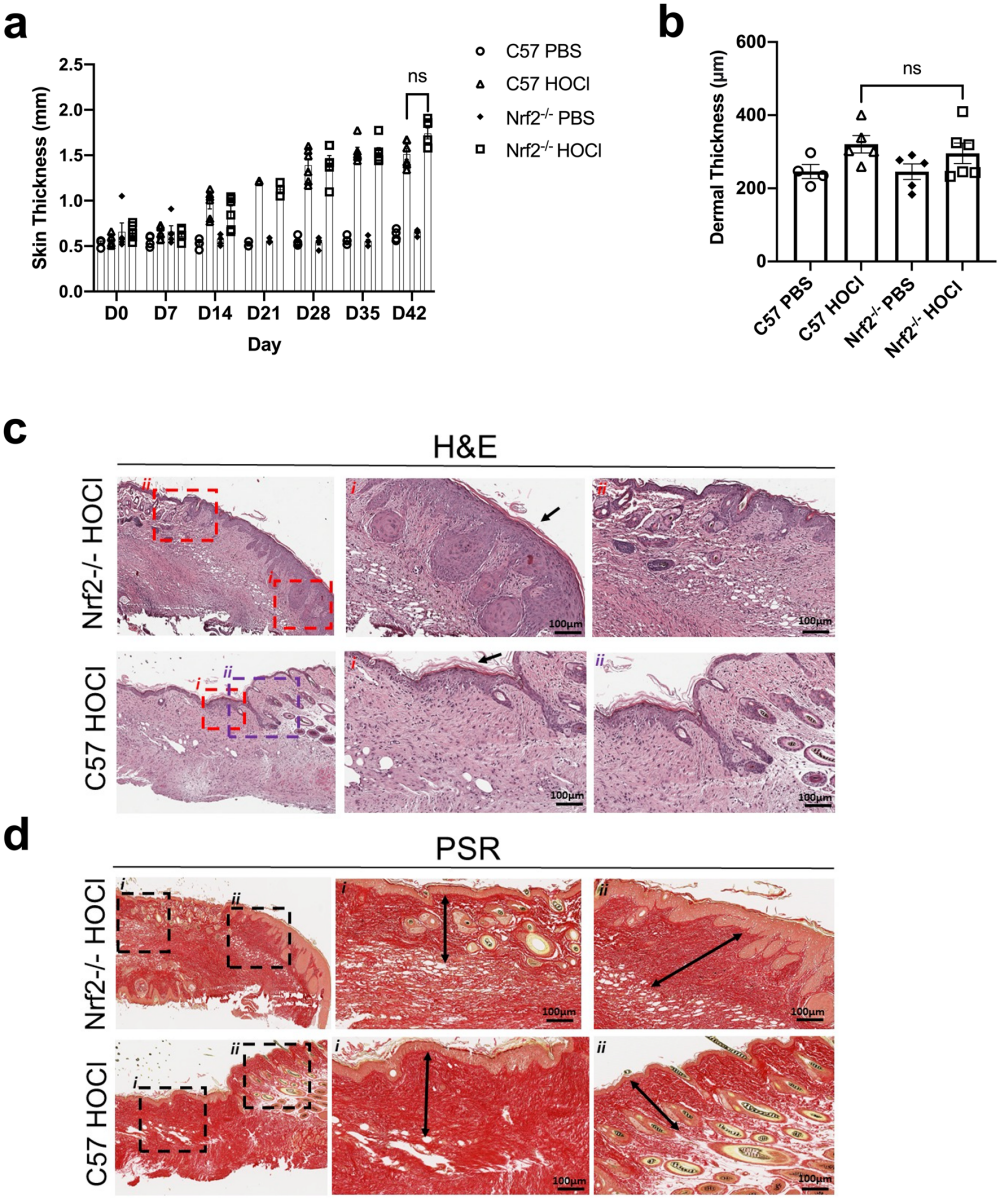
HOCl injections induce comparable skin fibrosis in Nrf2^−/−^ and WT control animals. (**a**) Skin thickness in mice increases over the course of 6 weeks of HOCl injections (*n* = 4–6). (**b**) Average measurements of dermal thickness (*n* = 4–6). Representative images of (**c**) H&E and (**d**) PSR staining of Nrf2^−/−^ and C57 HOCl-treated mice. H&E sections of both Nrf2^−/−^ and C57 mice show epidermal hyperplasia and hyperkeratosis indicated by the black arrow (H&E staining, panels *i*) and loss of adipose tissue/appendages in certain areas with preservation of them in adjacent areas (H&E staining, panels *ii*). Black arrows in the PSR-stained sections show representative dermal measurements. Data are represented as mean ± SEM from 2 experiments. Data were analyzed using Two-way ANOVA (**a**) or Mann–Whitney *U* test (**b**). *ns* not significant.

Next, we evaluated whether Nrf2 deficient mice had increased skin fibrosis when exposed to HOCl compared to C57BL/6 PBS-injected mice. Over a period of 6 weeks, no significant difference in skinfold thickness was observed (Nrf2^−/−^ HOCl vs C57 HOCl on day 42, mm: 1.74 ± 0.06 vs 1.51 ± 0.06) (Fig. 6a). Histology of Nrf2^−/−^ HOCl and C57 HOCl were comparable (Fig. 6c,d). Consistent with these findings, on day 42 there was no difference between the dermal thickness of Nrf2^−/−^ HOCl and C57BL/6 HOCl mice (Nrf2^−/−^ HOCl vs C57 HOCl, mm: 295.80 ± 28.09 vs 320.50 ± 23.64) (Fig. 6b).

### HOCl injections neither induce lung histologic abnormalities nor impair lung function in Nrf2^−/−^ mice

**Figure 7:**
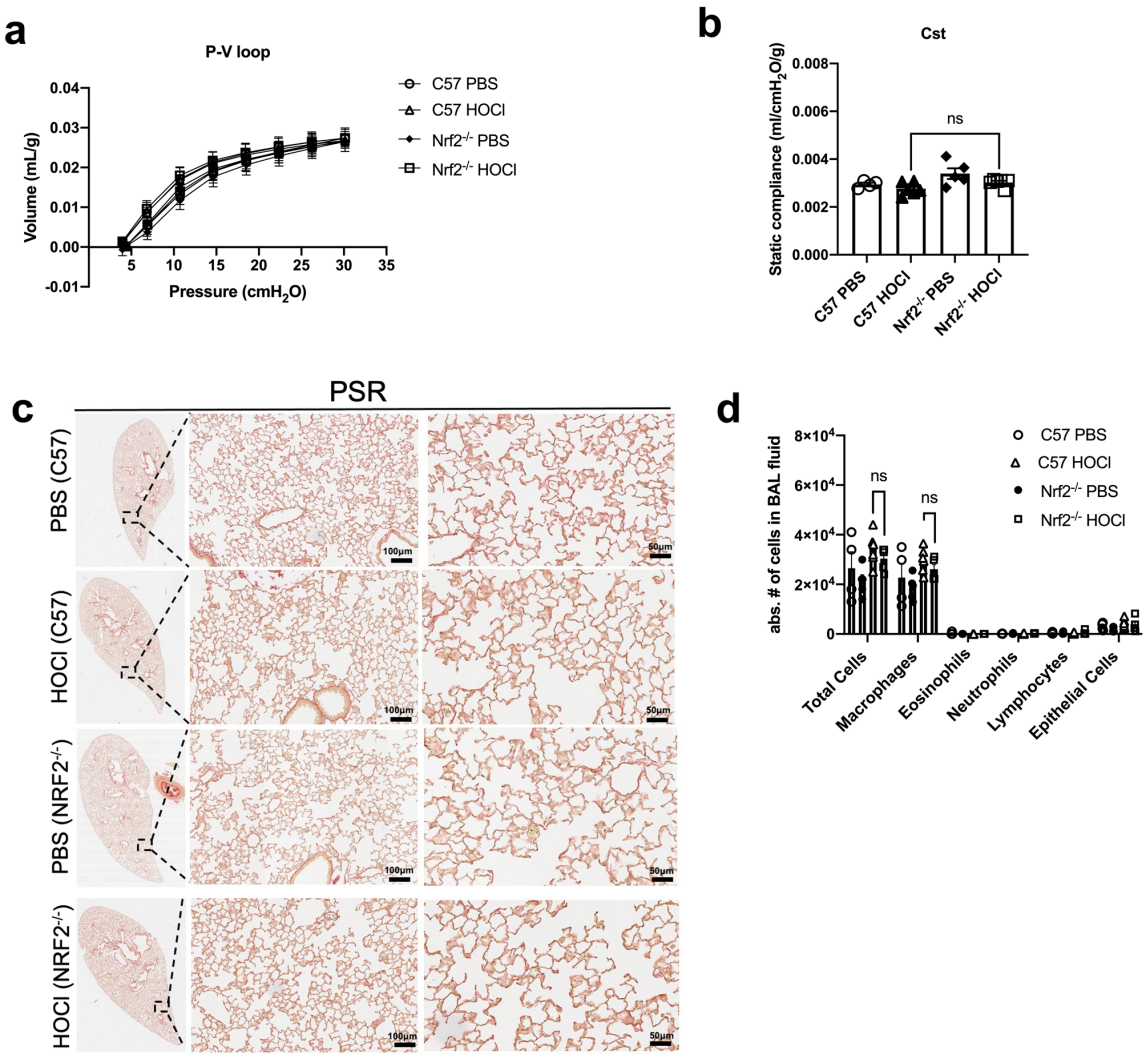
HOCl injections neither impair lung function nor induce lung fibrosis or inflammation in Nrf2^−/−^ mice. (**a**) Mean pressure–volume (P–V) loops for C57BL/6 and Nrf2^−/−^ mice injected with either PBS or HOCl. (**b**) Static lung compliance (C_st_) (*n* = 4–6). Micrographs of (**c**) PSR-stained lung sections (n = 4–6). Images were obtained to show a low magnification of the lung and at × 20 and × 40 magnification (from left to right). Scale bar: 100 μm (× 20 magnification) and 50 μm (× 40 magnification). (**d**) Total and differential cell counts in BALF of C57BL/6 and Nrf2^−/−^ mice injected with HOCl or PBS (n = 4–6). Data are represented as mean ± SEM from 2 experiments. Data were analyzed using Two-way ANOVA (**a**, **d**) or Mann–Whitney *U* test (**b**). *ns* not significant.

Lung function measurements showed no difference in respiratory system compliance between Nrf2^−/−^ and wild type C57BL/6 HOCl-injected mice (Nrf2^−/−^ vs C57: 3.03 × 10^–3^ ± 6.84 × 10^–5^ vs 2.76 × 10^–3^ ± 1.07 × 10^–4^ ml/cmH_2_O/g) (Fig. 7a,b). Lung histology also confirmed the absence of thickened alveolar septa (Fig. 7c). No excess collagen was detected in PSR-stained slides (Fig. 7c). These results indicate that Nrf2^−/−^ mice were not susceptible to HOCl-induced lung fibrosis.

### HOCl injections did not induce lung inflammation in Nrf2^−/−^ mice

It was previously shown that the Nrf2 pathway is involved in attenuating lung inflammation in bleomycin-induced injury^33,34^. Therefore, in the absence of Nrf2, inflammatory responses are expected to be increased. We assessed whether Nrf2^−/−^ mice had increased airway inflammation by analyzing total and differential cell counts in BALF. Compared to wild type C57BL/6 HOCl mice, total cells were not increased in Nrf2^−/−^ HOCl mice (C57 HOCl vs Nrf2^−/−^, absolute number of cells: 34,167 ± 2626 vs 30,400 ± 2064) (Fig. 7d). Moreover, there were no differences in the number of macrophages, eosinophils, neutrophils, and lymphocytes between these two groups. These data show that HOCl-generated oxidative stress does not result in the development of pulmonary inflammation following intradermal HOCl administration.

## Discussion

Several animal models of SSc have been proposed in the literature. Among these models is the mouse intradermally injected with HOCl^9,11^. We performed an in-depth characterization of the proposed HOCl-SSc model, a model strongly favoured in the literature. Over the course of 6 weeks, skinfold, and dermal thickness of HOCl mice increased approximately 2- and threefold, respectively. Skin histology was characterized by patchy areas of fibrosis adjacent to unaffected skin. Consistent with these observations, the skin of HOCl-treated mice showed upregulated expression of fibrosis-related genes including *Col1a1*, *Col3a1*, and *Acta2*. Similarly, BLM-MP mice showed evidence of skin fibrosis. However, there was a striking lack of changes in the lungs; there were no differences in static compliance, histology, expression of pro-fibrotic genes, and immune cell compositions between PBS and HOCl-treated animals. This contrasted the findings of the BLM-MP model which showed lung inflammation and evidence of patchy fibrosis on histology. Micro-CT abnormalities and significant lung function impairment was also observed in BLM animals. Additionally, neither AOPPs nor anti-Scl70 antibodies were elevated in the sera of HOCl-treated mice. Furthermore, to test the hypothesis that mice susceptible to oxidative damage show enhanced lung fibrosis following HOCl administrations, experiments were conducted using the Nrf2^−/−^ mouse. Nrf2^−/−^ mice that received HOCl injections had comparable skin fibrosis, lung function, lung histology and BALF cellularity to Nrf2^−/−^ controls.

Several studies have reported that HOCl induces a gradual increase in skinfold and dermal thickness within a range similar to our results^12,35^. H&E- and MT-stained skin sections from HOCl animals showed a pattern of fibrotic injury characterized by spatial heterogeneity, epidermal hyperplasia and hyperkeratosis, consistent with prior reports^10,12,35^. These changes are also seen in SSc patients^36^. In some sections fibrosis was predominantly localized to the deep reticular dermis rather than to the papillary dermis^35^. It is important to note that the HOCl-induced skin manifestations were only observed in the lower back of the animal where injections were administered and not elsewhere, indicating a lack of a generalized fibrotic cutaneous response. The BLM-induced skin lesions also produced a phenotype resembling SSc, however, the observed effect was not restricted to the site of the minipump.

Following 6 weeks of HOCl injections, the lung function of BALB/c mice remained unchanged which corresponded with the lack of morphological alterations in the lung. Similarly, HOCl injections failed to generate lung fibrosis in C57BL/6 mice. Our findings are in contrast to several studies that report evidence of pulmonary fibrosis using similar methods^10,12,13^. We also performed automated and manual image analysis, which was not previously conducted, on PSR-stained lung sections and no differences were detected between PBS- and HOCl-injected mice. Assessment of inflammatory cells in the BAL fluid did not show any evidence of an inflammatory response in HOCl-injected mice. Several technical factors such as inhomogeneous lung inflation resulting in areas of atelectasis and thick histological sections may suggest architectural abnormalities that could lead to erroneous interpretations of lung histology. Additional factors that could account for the discrepancies between our outcomes and published results include housing facility aspects (e.g. sterility, temperature, stressors, enrichments provided, etc.) and/or differences in mice purchased from different vendors.

Despite the lack of evidence for pulmonary injury, we characterized macrophage subsets in PBS and HOCl-treated mice, given their role in bleomycin-induced lung injury and fibrosis^37,38^. We addressed the three subsets, tissue resident alveolar macrophages (TR-AM), monocyte-derived alveolar macrophages (Mo-AM), and interstitial macrophages (IM)^37^. Under normal conditions TR-AM are the most abundant immune cell in the lung. Conversely, in the setting of lung inflammation, circulating monocytes are recruited to the lung via the activation of chemokine receptor 2 (CCR2) and differentiate into Mo-AMs^37^. Initially Mo-Ams drive the inflammatory and fibrotic response; however, during repair it is proposed that Mo-Ams can either differentiate into cells that phenotypically resemble TR-AM or undergo apoptosis^37^. Several groups reported that depletion of circulating monocytes by intratracheal administration of liposomal clodronate^39^ or using monocyte-chemoattractant protein-1 chemokine receptor knockout animals (CCR2^−/−^)^40^ attenuated bleomycin-induced lung injury. These findings show that monocytes contribute to facilitating the progression of lung fibrosis. It has been suggested that monocytes differentiate into Mo-Ams and in combination with TR-AM acquire a profibrotic phenotype^37^. Indeed, inducing cell death of Mo-AM ameliorates the severity of lung fibrosis^38^. Following 42 days of HOCl injections, an increase in the number of Mo-AM was expected given the pathobiologic role of Mo-Ams in fibrosis^38^. During the fibrotic phase of bleomycin-induced lung injury, a decrease in the number of interstitial macrophages and an increase in the number of Mo-AM has been reported^37^. This is likely attributed to the large inflammatory response induced by bleomycin which causes the loss of most macrophages present under homeostatic conditions (TR-AM and IM) and their replacement by Mo-AM. The absence of an inflammatory response in our HOCl model contrasts with a previous report which showed increased CD4 + and CD8 + T cells, and CD19 + B cells in the lung by immunohistochemical analysis^17^. Moreover, we did not observe an upregulation of profibrotic genes in HOCl-injected mice whereas the mRNA expression of *αSMA*, *TGF-β1*, and *collagen type I* and *III* were upregulated in previous studies^4,12^.

HOCl has been proposed to generate lung fibrosis through excess formation of reactive oxygen species (ROS) and the induction of oxidative stress. Specifically, oxidized serum proteins (i.e. AOPPs) were suggested to be involved in the propagation of oxidative stress from the skin to the lungs^9^. However, our experiments failed to demonstrated change in serum AOPPs. In the case of excess oxidative stress, there is an imbalance between the production of ROS and cellular antioxidant defense mechanisms. Since we did not observe elevated AOPPs following HOCl injections, we hypothesized that the ROS generated by HOCl are counteracted by physiological antioxidant defense mechanisms that maintain redox homeostasis. Nuclear factor erythroid 2-related factor 2 (Nrf2) is an important transcription factor for the activation of antioxidant pathways in the cell. Nrf2 protects against oxidative injury by binding to promoter sequences termed antioxidant response elements. This results in the induction of cytoprotective and antioxidant genes. The importance of Nrf2 in activating cytoprotective genes was sustained through studies showing that the induction of Nrf2 protected cells from ROS in different tissue types^41,42^. Other reports have shown that inactivation of Nrf2 exposed cells to increased oxidants leading to increased susceptibility to lung disease^33,43^. Nrf2^−/−^ mice treated with bleomycin had increased BAL leukocyte numbers, increased lung hydroxyproline levels, and more prominent histological fibrosis^33^. These data suggest that the Nrf2 antioxidant pathway is important in limiting bleomycin-induced lung fibrosis^33^. To test the hypothesis that deficient antioxidant responses increase the skin and lung toxicity of intradermal HOCl, we subjected Nrf2^−/−^ animals to daily HOCl injections. Nrf2^−/−^ animals injected with HOCl were comparable to C57BL/6 HOCl injected animals in their skin fold thickness, skin histology, dermal thickness, BAL cellularity, lung mechanics, and lung histology. Conversely, one paper reported an increase in skin thickness, collagen content in the skin and lungs, and exacerbated skin and lung pathology in Nrf2^−/−^ HOCl-treated animals^4^.

Previous papers have used commercial bleach at a concentration of 9.6% whereas we used bleach with a sodium hypochlorite concentration of 4.0%. However, this is unlikely to account for the discrepancies observed as the final concentration of the injected HOCl solution was the same as that reported by other groups. We also compared the effect of 4.0%, 10.0%, and 13.0% commercial bleach administered at the same final concentrations and did not observe any difference in lung function (Supplementary Fig. 5). We also tried to increase the administered concentration of the HOCl solution; however, these experiments were limited by the severe ulcers the mice developed.

In conclusion, the administration of HOCl intradermally following published protocols did not reproduce the putative lung fibrosis. Despite sensitizing the model to the potential development of fibrosis by studying Nrf2^−/−^ mice with defective antioxidant responses, there was no lung pathology identified by careful histological examination, quantification of lung collagen and lung function. The basis for the discrepancy between our pulmonary findings and those described in the literature is not clear. We cannot exclude the possibility that substrain differences in mice are present when procured from different sources. However, it should be noted that the lung phenotype in this model has not been previously characterized in detail from a functional standpoint. Prior publications do not report lung function measurements nor histological quantification of lung fibrosis, both of which can provide more accurate assessments of lung involvement. Studies have frequently relied upon the measurement of hydroxyproline, an indirect surrogate for collagen synthesis. While the HOCl-treated mouse may be used to model SSc-like skin fibrosis the fibrotic lesions are not generalized but restricted to the areas of skin directly affected by HOCl injections. In contrast, lung mechanics of SSc-ILD are recapitulated by the BLM-MP model. In contrast, lung mechanics, CT abnormalities, and some histological features of SSc-ILD are recapitulated by the BLM-MP model. Therefore, the HOCl model is at the very best inconsistent compared to the BLM-MP model of oxidative stress-induced lung fibrosis.

### Supplementary Information

Supplementary Figures.
