# Supplementary material for: Superiority of systemic bleomycin to intradermal HOCl for the study of interstitial lung disease
Source: Sci Rep. 2023 Nov 23;13:20577. doi: 10.1038/s41598-023-47083-y (PMC10667597; doi:10.1038/s41598-023-47083-y)
Supplement: Supplementary file 1 — Supplementary Figures. [file 41598_2023_47083_MOESM1_ESM.docx]

**Supplementary Figures**

**Title:** Superiority of systemic bleomycin to intradermal HOCl for the study of interstitial lung disease

**Authors:** Arina Morozan^†§^, Sydney Joy^†§^, Utako Fujii^†^, Richard Fraser^#^, Kevin Watters^#^, James G. Martin^†§&^, Inés Colmegna^§##^*^&^

**Affiliations**:

^†^Meakins Christie Laboratories, McGill University Health Centre and McGill University, Montreal, Quebec, Canada, H4A 3J1

^§^The Research Institute of the McGill University Health Centre, McGill University, Montreal, Quebec, Canada, H4A 3J1

^#^ Division of Pathology, McGill University Health Centre, Montreal, Quebec, Canada.

^##^ Division of Rheumatology, McGill University Health Centre, McGill University, Montreal, Quebec, Canada.

^&^ Joint senior authors.

Supplementary Figure 1

**b**

**a**


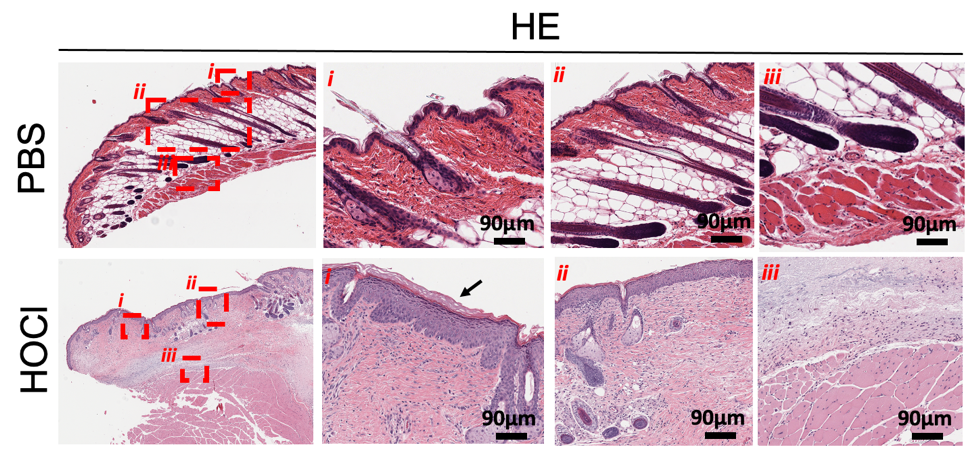


**c**


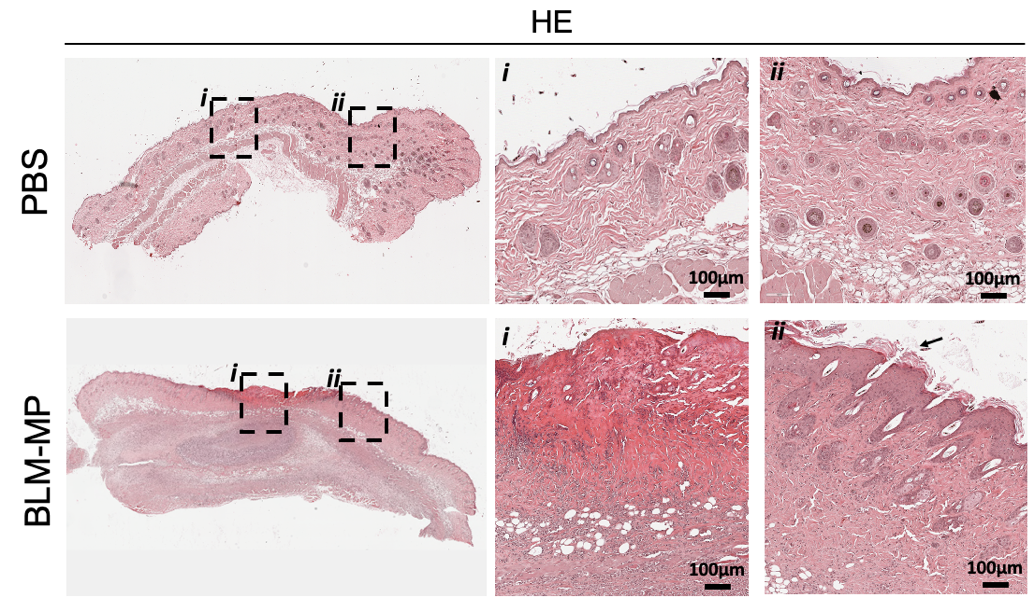


**Supplementary Figure 1.** **Skin fibrosis in HOCl and BLM-MP mice.** **a.** Mice skin thickness increased over the course of 6-weeks of HOCl injections. *n* = 11-14 mice per group. ** *p*=7.50 **×** 10^-3^, **** *p*< 1.00 **×** 10^-4^. Data are represented as mean ± SEM from 2-3 independent pooled experiments. Data were analyzed using two-way ANOVA. **b.** Representative skin images of H&E staining of PBS and HOCl-treated mice. Overview and ×20 magnification. Scale bar: 90 μm. H&E sections of HOCl mice show epidermal hyperplasia and hyperkeratosis (HOCl row, black arrow, *i*); loss of adipose tissue/appendages (*ii*); and inflammation extending into the superficial muscle layer (*iii*). **c.** Representative skin images of H&E staining of PBS and BLM-treated mice. Images show a skin overview (first micrograph in each row) and ×20 magnification. BLM-MP mice show an area of ulceration (panel *i*), epidermal hyperplasia and hyperkeratosis indicated by the black arrow (BLM-MP row, panel *ii*).

Supplementary Figure 2

**b**

**a**

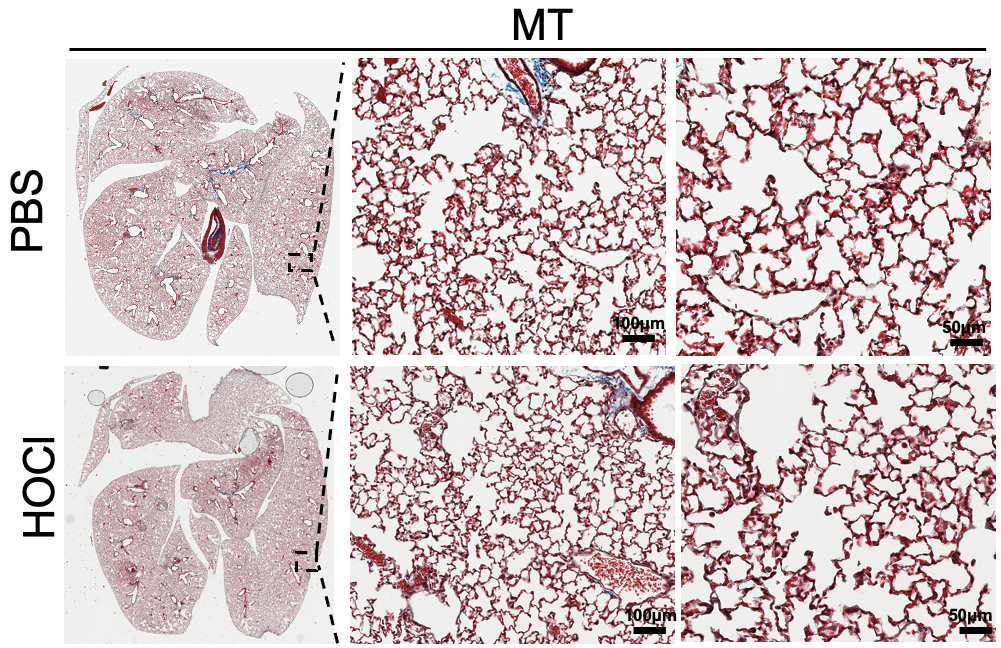
**
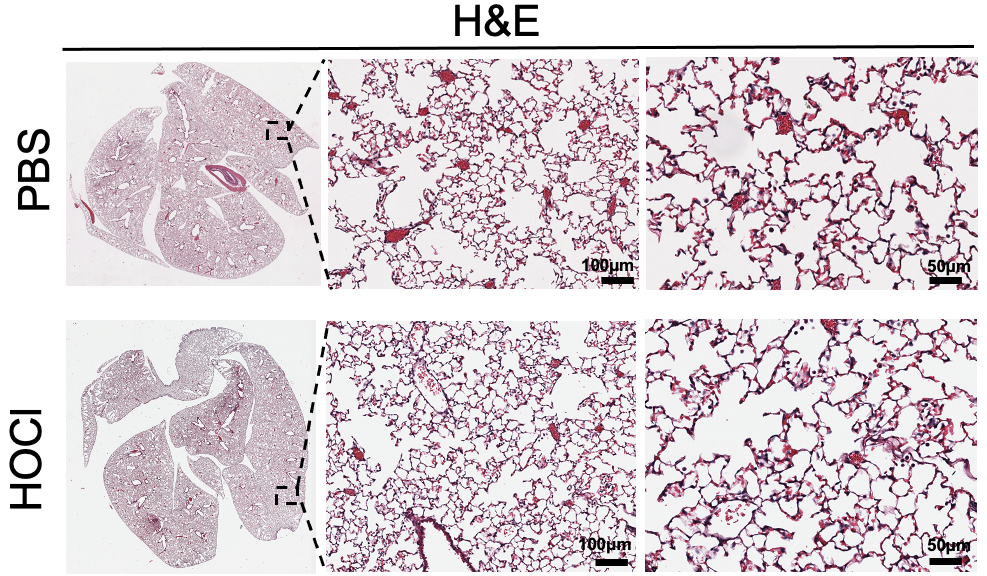
**

**d**

**c**

**Supplementary Figure 2. Intradermal HOCl injections (8 weeks) do not impair lung function or induce lung fibrosis. a.** Mean pressure-volume (P-V) loops for PBS and HOCl-injected mice. **b.** Static lung compliance (C_st_) (*n*=5-6). **c.** Micrographs of H&E and **(d)** MT-stained lung sections. Images show an overview of the lung and ×20 and ×40 magnification (from left to right). Scale bar: 100 μm (×20 magnification) and 50 μm (×40 magnification). Data are represented as mean ± SEM from one experiment. Data were analyzed using Two-way ANOVA (a) and Mann-Whitney U test (b). Abbreviations: ns: not significant.

Supplementary Figure 3


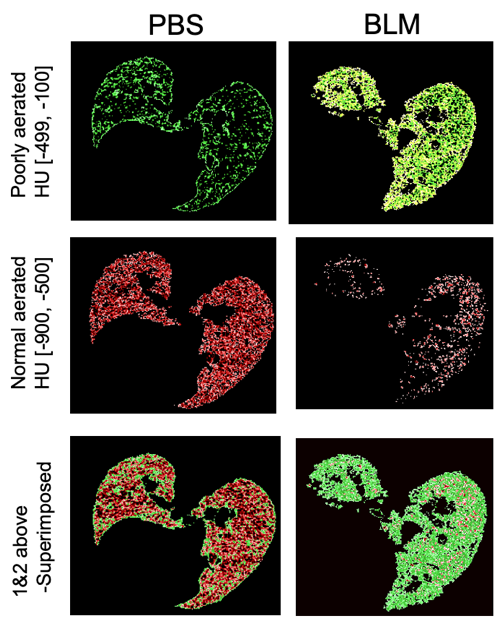

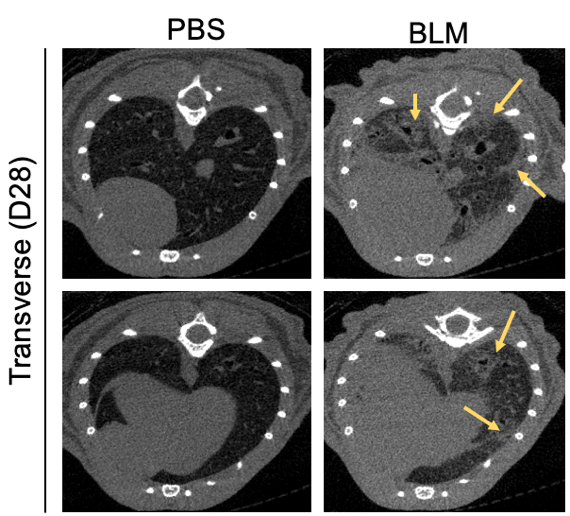


**e**

**a**

**f**

**d**

**c**

**b**

**Supplementary Figure 3. Osmotic minipump delivery of BLM increases lung density and the percent of poorly aerated tissue.** **a.** Representative micro-CT scans of PBS and BLM animals. Yellow arrows indicate areas of increased lung attenuation. Density of lung parenchyma in whole lung **(b)**, right lung **(c)** and left lung **(d)**. **e.** Micro-CT scans of BLM mice evaluated with the image analysis software AMIDE have increase in poorly- aerated tissue. **f.** Quantification of poorly aerated tissue according to Hounsfield units (HU) range assessed with the MATLAB software. *n*=3-6 mice per group, graphs show ± SEM. Two tailed t test, * *p*<0.05

Supplementary Figure 4


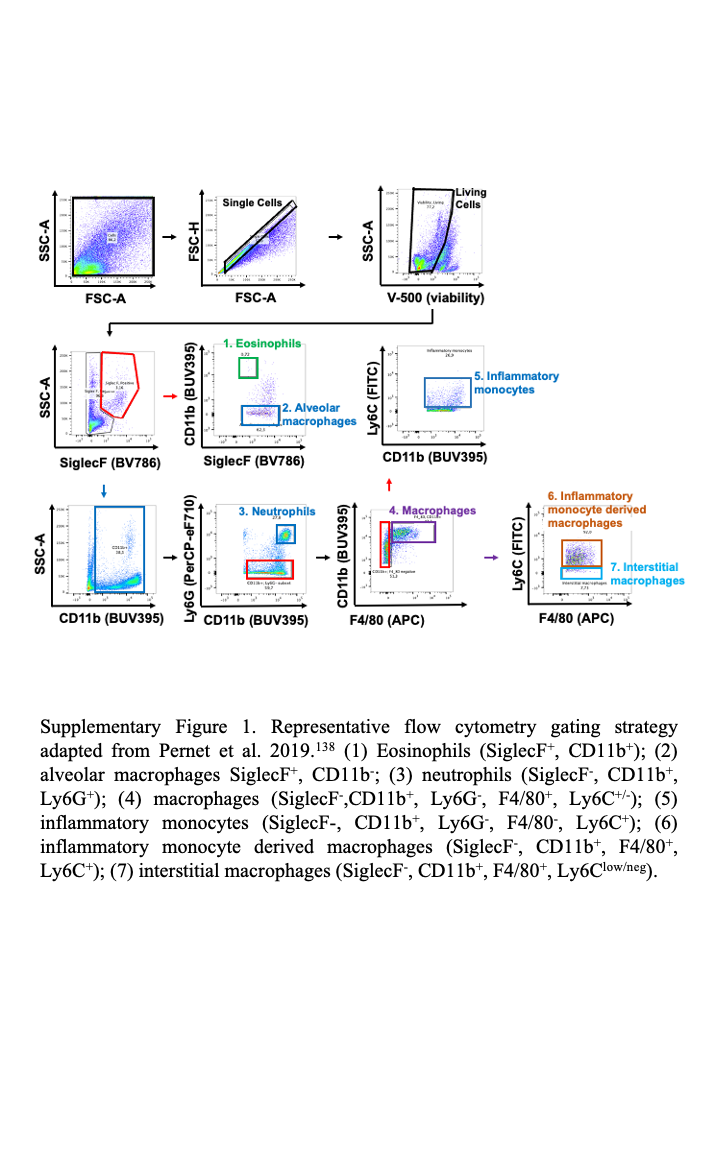


**Supplementary Figure 4. Representative flow cytometry gating strategy adapted from Pernet et al. 2019.**^138^ (1) Eosinophils (SiglecF^+^, CD11b^+^); (2) alveolar macrophages SiglecF^+^, CD11b^-^; (3) neutrophils (SiglecF^-^, CD11b^+^, Ly6G^+^); (4) macrophages (SiglecF^-^,CD11b^+^, Ly6G^-^, F4/80^+^, Ly6C^+/-^); (5) inflammatory monocytes (SiglecF-, CD11b^+^, Ly6G^-^, F4/80^-^, Ly6C^+^); (6) inflammatory monocyte derived macrophages (SiglecF^-^, CD11b^+^, F4/80^+^, Ly6C^+^); (7) interstitial macrophages (SiglecF^-^, CD11b^+^, F4/80^+^, Ly6C^low/neg^).

Supplementary Figure 5

**b**

**a**

**Supplementary Figure 5. Different initial concentrations of commercial bleach DO (does) not affect lung compliance.** **a.** Mean pressure-volume (P-V) loops of mice injected with bleach containing initial sodium hypochlorite concentrations of 4%, 10%, and 13% (the final concentration was the same for all the mice, 0.096%). **b.** Static lung compliance. Data are represented as mean ± SEM from 2 independent pooled experiments. P-V loops and static compliance were analyzed using repeated measures two-way ANOVA and one-way ANOVA, respectively, with Bonferoni post-hoc test.
